# Supplementary material for: Content-rich biological network constructed by mining PubMed abstracts
Source: BMC Bioinformatics. 2004 Oct 8;5:147. doi: 10.1186/1471-2105-5-147 (PMC528731; doi:10.1186/1471-2105-5-147)
Supplement: Additional File 5 — The original Chilibot query results of the term "long-term potentiation (LTP)" and 22 other terms, limiting the latest references analyzed to the years 1990, 1995, 2000, and 2004. [file 1471-2105-5-147-S5.bz2 › chilibotAdditionalFile5/ltp2000/html/PLC_PI-3K.html]

 


 **PLC** and **PI-3K** 
  
Found 79 abstracts in PubMed,  **30 abstracts were retrieved and analyzed**.  


---

 Search Google  |
 PDF files only 
|  EDU domain only 

---

**Interactive relationship** (e.g. stimulation, inhibition, etc)

- In contrast, the increase in chemotactic response seen upon overexpression of  **PLC**  gamma1 was not inhibited by the PI3 kinase  [ **PI-3K** ]  inhibitor LY294002.  Ref: 10419537 J Biol Chem, 1999
- Inhibition of PI 3 kinase  [ **PI-3K** ]  by wortmannin or LY294002 abolished platelet secretion and aggregation, as well as phospholipase C  **PLC**  activation, indicating a role of this lipid kinase in the early phase of platelet activation.  Ref: 9733717 J Biol Chem, 1998
- Specific inhibitors of protein kinase C, bisindolylmaleimide GF109203X, and phosphatidylinositol 3 kinase PI3 kinase  [ **PI-3K** ] , LY294002, did not affect the activation of  **PLC**  gamma1.  Ref: 10419537 J Biol Chem, 1999
- in contrast,  **PLC**  inhibitor U 73122 did suppress adhesion, suggesting the possibility that  **PLC** ... PKC, or PKA, may be involved in this process.  Ref: 9682000 Cell Immunol, 1998
- Indeed, tyrosine phosphorylation of  **PLC**  gamma, which has been demonstrated to bind to pY1021... was reduced in APS transformants.  Ref: 9989826 Oncogene, 1999

**Parallel relationship** (e.g. studied together, co-existance, homology, etc.)

- In this study, we investigated the effect of PDGF AA on cell migration, PI 3 kinase  [ **PI-3K** ]  and phospholipase C  **PLC**  activation, and the role of these two enzymes in mediating biological responses in these cells in response to all three isoforms.  Ref: 10720944 Kidney Int, 2000
- Fibroblast growth factor 2 uses  **PLC**  gamma1 for cell proliferation and PI3 kinase  [ **PI-3K** ]  for alteration of cell shape and cell proliferation in corneal endothelial cells.  Ref: 9808840 Mol Vis, 1998
- Using immunoprecipitation, PI3 kinase  [ **PI-3K** ]  and tyrosine phosphorylated  **PLC**  gamma1 and SHC were shown to be associated with tyrosine phosphorylated TrkB in response to both BDNF and NT 4.  Ref: 10486198 Exp Neurol, 1999
- Mutational analysis indicates that coactivation of phospholipase C gamma  **PLC**  gamma and phosphoinositide 3 kinase PI3 kinase  [ **PI-3K** ]  by specific cytoplasmic domains of TrkA is essential for triggering chemoattraction of the growth cone in an NGF gradient.  Ref: 10402200 Neuron, 1999
- Taken together, these data demonstrate that all three isoforms of PDGF significantly stimulate  **PLC**  gamma 1 and PI 3 kinase  [ **PI-3K** ] , two enzymes necessary for both DNA synthesis and directed migration.  Ref: 10720944 Kidney Int, 2000
- The nascent models highlight the roles of various intracellular signaling pathways including phospholipase C gamma  **PLC**  gamma, phosphatidylinositol PI 3 kinase  [ **PI-3K** ] , mitogen activated protein MAP kinase, and actin cytoskeleton related events.  Ref: 10547668 Adv Cancer Res, 2000
- depending on the cellular context, the PI3 kinase  [ **PI-3K** ]  pathway or the  **PLC**  gamma1 pathway may dominate.  Ref: 10419537 J Biol Chem, 1999
- These results, together with the effects of pharmacological inhibitors, support the notion that there are common cytosolic signaling pathways for two separate groups of guidance cues, one of which requires coactivation of  **PLC**  gamma and PI3 kinase  [ **PI-3K** ]  pathways.  Ref: 10402200 Neuron, 1999
- FAK was also associated with signaling molecules such as  **PLC**  gamma and PI3 kinase  [ **PI-3K** ]  through c Src.  Ref: 11095944 Biochem Biophys Res Commun, 2000
- Activation of  **PLC**  and PI 3 kinase  [ **PI-3K** ]  by PDGF receptor alpha is not sufficient for mitogenesis and migration in mesangial cells.  Ref: 10720944 Kidney Int, 2000
- The authors investigated the roles of PI3 kinase  [ **PI-3K** ]  and  **PLC**  gamma in stimulation by Steel Factor SLF through c Kit.  Ref: 11090054 Blood, 2000
- SHC,  **PLC**  gammal, Ras, PI 3 kinase  [ **PI-3K** ]  and SNT.  Ref: 10467403 Oncogene, 1999
- the obtained data suggest that an activation of alpha1A adrenoceptors, presence in WAT, by agonist and or neurotransmitter may increase the glucose uptake via  **PLC**  PKC pathway and the activation of PI 3 kinase  [ **PI-3K** ] .  Ref: 11111846 Auton Neurosci, 2000
- However, activation of alpha receptor by PDGF AA with a subsequent increase in  **PLC**  and PI 3 kinase  [ **PI-3K** ]  activities is not sufficient to induce these biological responses in mesangial cells.  Ref: 10720944 Kidney Int, 2000
- Thus, we examined the interactions of various SH2 containing molecules like  **PLC**  gamma1, rasGAP, p85 subunit of PI3 kinase  [ **PI-3K** ] , Src, Fyn, Crk, Nck, Grb2 and Shc with HEK2 using in vitro binding assays, immunoprecipitations and yeast Two Hybrid assays.  Ref: 9674711 Oncogene, 1998
- In addition, villin was found to associate with the 85 KDa subunit p85 of phosphatidylinositol PI 3 kinase  [ **PI-3K** ] , concomitant with  **PLC**  gammal, in the cytoskeletal fraction of Na P i cotransport up regulated cells.  Ref: 10949911 Mol Med, 2000
